# Imaging Subthreshold Voltage Oscillation With Cellular Resolution in the Inferior Olive *in vitro*

**DOI:** 10.3389/fncel.2020.607843

**Published:** 2020-12-14

**Authors:** Kevin Dorgans, Bernd Kuhn, Marylka Yoe Uusisaari

**Affiliations:** ^1^Neuronal Rhythms in Movement Unit, Okinawa Institute of Science and Technology Graduate University, Okinawa, Japan; ^2^Optical Neuroimaging Unit, Okinawa Institute of Science and Technology Graduate University, Okinawa, Japan

**Keywords:** acute slice preparation, *in vitro*, voltage-sensitive dye, network activity, inferior olive, subthreshold oscillations, voltage imaging, wide-field fluorescence imaging

## Abstract

Voltage imaging with cellular resolution in mammalian brain slices is still a challenging task. Here, we describe and validate a method for delivery of the voltage-sensitive dye ANNINE-6plus (A6+) into tissue for voltage imaging that results in higher signal-to-noise ratio (SNR) than conventional bath application methods. The not fully dissolved dye was injected into the inferior olive (IO) 0, 1, or 7 days prior to acute slice preparation using stereotactic surgery. We find that the voltage imaging improves after an extended incubation period *in vivo* in terms of labeled volume, homogeneous neuropil labeling with saliently labeled somata, and SNR. Preparing acute slices 7 days after the dye injection, the SNR is high enough to allow single-trial recording of IO subthreshold oscillations using wide-field (network-level) as well as high-magnification (single-cell level) voltage imaging with a CMOS camera. This method is easily adaptable to other brain regions where genetically-encoded voltage sensors are prohibitively difficult to use and where an ultrafast, pure electrochromic sensor, like A6+, is required. Due to the long-lasting staining demonstrated here, the method can be combined, for example, with deep-brain imaging using implantable GRIN lenses.

## 1. Introduction

Investigating neuronal activity on network and single-cell level with the use of fluorescent voltage indicators has in recent years been regaining popularity. While fluorescent calcium imaging remains the most common approach due to the ease of use, it is impractical for experiments involving subthreshold activity such as synaptic potentials or, in case of the inferior olive (IO), subthreshold oscillations (STOs) of the membrane voltage.

The IO STOs are regular, sinusoidal fluctuations of the membrane voltage, ranging between 3 and 12 Hz in frequency and occasionally reaching up to 15 mV peak-to-peak amplitudes but most commonly ranging between 2 and 10 mV (Llinás and Yarom, 1986; Chorev et al., 2007; Khosrovani et al., 2007). Importantly, while they are dramatically smaller in amplitude on single-cell level than other commonly-studied oscillatory phenomena in mammalian brains that reflect synchronization of suprathreshold action potentials (such as gamma or theta oscillations; Cunningham et al., 2004; Hummos and Nair, 2017), the IO STOs are considered a key component for input integration and subsequent complex spike activity in the cerebellum (Jacobson et al., 2009; Llinás, 2009; Negrello et al., 2019). They involve network-level interactions among gap-junction coupled neurons rather than being solely driven by intra-cellular dynamics (Manor et al., 2000; Loewenstein et al., 2001; Long et al., 2002; Leznik and Llinás, 2005; Placantonakis et al., 2006; de Gruijl et al., 2012; Lefler et al., 2014). What brings importance to the need to resolve spatial arrangement of IO STOs is their proposed key role in timing and modulating action potential generation. IO spikes are the source of synchronous cerebellar complex spike activity that in turn underpin the construction of overall cerebellar output (Wise et al., 2010; Streng et al., 2018; Tang et al., 2019; Arlt and Häusser, 2020).

The interest in cerebellar synchronicity leads to the necessity of monitoring the STOs in populations of IO neurons in order to examine the underlying physiological mechanisms, as single-cell patch-clamp recordings will fail to deliver the whole picture of the dynamics involved. While calcium imaging is often the method-of-choice for examining network activity and generation of STOs relies on interactions between low- and high-threshold calcium currents (Chorev et al., 2008; Park et al., 2010), it is unclear if calcium imaging approaches could be utilized to monitor STOs as the calcium concentration changes might be too low to be resolved.

Voltage imaging has been successfully used to examine subthreshold voltage events in single cells (Jin et al., 2012; Popovic et al., 2015; Kwon et al., 2017; Roome and Kuhn, 2018, 2020). Such works require a high SNR that is challenging to be reached using genetically encoded voltage indicators (GEVIs; Milosevic et al., 2020) that allow targeting of the cell membrane in specific neuronal classes. Instead, they most commonly employ organic voltage-sensitive dyes (VSDs, such as JPW1114, Antić and Zečević, 1995) introduced into the examined neuron by means of pressured micro-injection (Antić and Zečević, 1995), a patch pipette (Canepari et al., 2013; Willadt et al., 2014), or electroporation (Roome and Kuhn, 2018), thereby limiting the source of signals to the investigated neuron. This approach is not, however, suitable for exploring circuit-wide subthreshold activity because neurons have to be individually labeled.

For voltage imaging of subthreshold activity on single-cell and network level, we set out to establish a method employing ANNINE-6plus (A6+), a highly lipophilic, purely electrochromic voltage sensor dye (Fromherz et al., 2008; Roome and Kuhn, 2018, 2019). A6+ exhibits superior voltage sensitivity and tissue-independent linearity of voltage responses with nanosecond-scale kinetics that should allow reliable monitoring of the IO subthreshold voltage fluctuations. However, A6+'s strong lipophilicity that is essential for a pure electrochromic behavior and reliably high sensitivity (0.5% per mV, Kuhn and Roome, 2019) poise difficulties in delivering it in a homogenous and broad manner to brain slices. Previous works (such as Eichhoff et al., 2008; Cameron et al., 2016; Tominaga et al., 2019) that have used DMSO-dissolved dyes commonly use bath or bolus loading of an acutely prepared brain slice. These methods deliver a high concentration of dye into the structure of interest. However, with A6+/DMSO bath or bolus loading, the major component of fluorescence will originate from the slice surface which contains the most damaged cells or cut off processes or near the injection site. Importantly, DMSO disturbs neuronal activity to the extent that circuit function may be compromised. This is especially problematic when examining phenomena such as the IO STOs that depend on fine balancing of interactions among gap junction-coupled neurons.

Here, we describe a modification for the method for introducing A6+ into the mouse inferior olive. The method involves injection of the dye into the IO in a living mouse up to 7 days prior to acute *in vitro* slice experimentation. The resulting labeling is more wide-spread and homogenous than with bolus loading, allowing better signal resolution across hundreds of micrometers. Additionally, somata are saliently brighter labeled. Therefore, we are able to monitor IO STOs, both at network as well as single-cell resolution in whole-field imaging mode. Importantly, we demonstrate that IO STOs can be recorded in single-trial manner without stimulation-triggered averaging as had been done earlier. The method can be modified for use with other brain structures, and further improvements in spatiotemporal resolution can be obtained using better optical elements, such as confocal, 2-photon imaging (Kuhn et al., 2004), or computer-generated holography (Tanese et al., 2017).

## 2. Materials and Methods

All animal experiments were performed in accordance with guidelines approved by the Okinawa Institute of Science and Technology Graduate University Institutional Animal Care and Use Committee (IACUC) in an Association for Assessment and Accreditation of Laboratory Animal Care (AAALAC International) accredited facility.

### 2.1. *In vivo* Injection of ANNINE-6plus Into the Inferior Olive

To prepare 100 μg/ml A6+ final solution, we added 40 μl of pure DMSO to 400 μg of A6+ (MW 717.6) and sonicated the suspension during 15 min at 35°. We progressively added ultrapure water and sonicated the suspension repetitively to obtain a transparent stock solution of 500 μl with 800 μg/ml A6+ (1.1 mM) and 8% DMSO. The stock solution is stored at −20°and diluted in PBS around 20 min before the injection to obtain the final solution of 100 μg/ml A6+ (140 μM) in 1% DMSO. 3% DMSO solutions were obtained by diluting A6+ stock solution in PBS, and adding 2% DMSO 20 min before experiment.

Postnatal (P) 40-55 c57bl/6j male mice (CLEA Japan, Shizuoka) were anesthetized with 5% isofluorane for induction (SomnoSuite, Kent Scientific, USA) and maintained in deep anesthetic state on a heat pad (38°C) attached to a stereotaxic frame (Neurostar, Tübingen, Germany) with 1.5% isofluorane. Before commencing the surgery, the skin covering skull was locally anesthetized with application of Xylocaine gel (Xylogel, gel 2%, Aspen Japan, JP) and the mice were administered 200 μl physiological saline (NaCl 0.93%) subcutaneously to prevent dehydration. The skin over the posterior skull was incised with a small scalpel blade and a small craniotomy was drilled bilaterally near the midline, 6.2 mm caudal to Bregma. One hundred nanoliters of A6+ solution was injected bilaterally into the IO nuclei (stereotactic coordinates relative to Bregma: antero-posterior, −6.2 mm; medio-lateral, ±0.42 mm; dorso-ventral, 6.7 mm; 100 nl/min) at 4 vertical locations 50 μm apart (from 6.7 to 6.5 mm dorso-ventral). Left and right IO received A6+ diluted in 1 and 3% DMSO, respectively. After injection was complete, the injection pipette was slowly withdrawn, the skin above the craniotomy was closed with superglue, and 200 μl of the anti-inflammatory drug carprofen (5 mg/kg; Rimadyl, Zoetis, US) was subcutaneously injected.

### 2.2. Sparse Labeling of IO Neurons With eGFP

In a subset of animals, IO neurons were sparsely labeled with eGFP for visualization of their soma shapes. Following the same surgical procedures as for A6+ injection, we injected bilaterally 400 nl of a mix of 2 viral constructs, AAV9.CamKII0.4.Cre.SV40 (AddGene viral prep 105558-AAV9) and AAV9.CAG.Flex.eGFP.WPRE.bGH (AddGene viral prep 51502-AAV9) in a ratio of 1:1; diluted 1:50 each, at the speed of 40 nl/min. After 6 weeks of transfection, the animals were transcardially perfusion-fixed (see section 2 below). pENN.AAV.CamKII 0.4.Cre.SV40 was a gift from James M. Wilson (Addgene viral prep # 105558-AAV9; http://n2t.net/addgene:105558; RRID:Addgene_105558), AAV pCAG-FLEX-EGFP-WPRE was a gift from Hongkui Zeng (Addgene viral prep #51502-AAV9; http://n2t.net/addgene:51502; RRID:Addgene_51502) (Oh et al., 2014).

### 2.3. Brain Fixation With Transcardial Perfusion and Slice Mounting

After appropriate staining or transfection periods, mice were transcardially perfusion-fixed with 50 ml of 4% para-formaldehyde (PFA) in phosphate-buffered saline (PBS; pH 7.4). The brains were extracted, post-fixed in 4% PFA overnight and washed in PBS before sectioning the brainstem region containing the IO (50 μm coronal sections) with a vibrating microtome (Campden Instruments, 5100MZ-plus Vibrotome). The sections were mounted with Vectashield with DAPI (VectorLabs, CA) or Prolong Glass (ThermoFisher Scientific, MA) and coverslipped with # 1.5 glass (Harvard Apparatus, US).

### 2.4. Confocal Imaging

Confocal image stacks were acquired from the IO slices with a Zeiss LSM 880 (Zeiss, Germany) with 20x (NA:0.8, Zeiss) or 40x objectives (NA:1.4, Oil, Zeiss). The low-magnification stacks of 40μm depth were acquired (field of view 450 × 450 μm, 1024 × 1024 pixel, pixel size 0.44μm, voxel depth 0.76 μm) with the 20x objective. Stack were acquired with 0.68 μm z-step image-series over 20 μm depth and a succession of 2 line averages with 16.38 μs pixel dwell time per pixel. Also, high-magnification images of 107 × 107 μm field of view were taken (pixel size 0.1 μm) with 4 frame averaging. A6+ was excited at 488 nm with a 25 mW Argon laser (laser power after the objective: less than 1 mW), and fluorescence was collected in the 530–620 nm range. DAPI was illuminated with a 405 nm diode and imaged in the 410–450 nm range. eGFP was excited at 488 nm with the Argon laser and images were acquired in the 500–550 nm wavelength band. In order to compare the intensity between the different animals, illumination and acquisition settings were saved and identically used for all acquisitions.

Mesoscale spread of A6+ in IO tissue as well as internalization of A6+ by IO neurons was assessed using *Radial Profile Angle* plugin from **FIJI** software (ImageJ, U. S. National Institutes of Health, USA, Schindelin et al., 2012). Cell centers were determined from a central DAPI staining point and radial profile calculated on circles of 20μm radius. Cells were picked within the area of 100–400 μm distance around the injection center. Histograms were normalized to maximal intensity.

### 2.5. Acute Slice Preparation and *in vitro* Voltage Imaging

Coronal brainstem slices of 300 μm thickness were prepared from the mice injected with A6+. The method (Huang and Uusisaari, 2013; Ankri et al., 2014; Eguchi et al., 2020) uses gassed and warmed (5% CO_2_, 95% O_2_; 37°C) standard physiological solution (SPS; composition: 126 mmol NaCl, 10 mmol glucose and 26 mmol NaHCO_3_, 3.4 ml KCl, 1.2 ml KH_2_PO_4_, then 1.3 ml MgSO_4_ (1M), 2 mL CaCl_2_ (1M); pH to 7.2–7.3). The same solution was used for slice recovery and the incubation chamber during the experiment. After extracting the brainstem from the skull, 4 coronal brainstem slices of 300 μm were obtained with a vibrating microtome (7000 smz-2, Campden Instruments, UK) equipped with ceramic blades (38 × 7 × 0.5 mm ceramic blades, model 7550-1-C, Campden Instruments, UK) at low slicing speed (0.01 mm/s). After an 1-h recovery period, slices are transferred to a submerged-type recording chamber, continuously perfused with warm (34°C) SPS, under an upright Olympus microscope (BXW51, Olympus, JP). Slices were viewed with 5x (NA:0.1, MPLN5X, Olympus) and 60x (NA:1.0, LUMPLFLN60XW, Olympus) objectives. To excite A6+, a 488 nm wavelength laser source (3W custom 488 nm laser, Coherent, USA) was used, with a typical power measured under the objectives in the range of 1.5 6.5 mW. Emitted fluorescence was high-pass filtered at 561 nm (RazorEdge ultrasteep long pass edge filter, Semrock, NY). Fluorescence images were acquired at 40–120 frames per second (fps), 256 × 256 pixel resolution (pixel size: 17.86 and 1.22 for 5x and 60x objectives, respectively) with a CMOS-based MiCAM03 imaging system (BrainVision, Japan).

### 2.6. Electrophysiology

Whole-cell patch-clamp recordings were performed during imaging experiments. Inferior olive neurons were located using infrared DIC optics, first at 5x magnification, then neurons individually selected at 60x with a sCMOS camera (Zyla4.2, Andor, UK). Cells were patched with 10 MOhm borosilicate pipettes (un-polished quartz capillaries with filament and with outer and inner diameters of 1.5 mm and 0.86 mm, respectively; BF-150-86-10, Sutter Instrument, USA) filled with intracellular solution containing 4 mM NaCl, 0.001 mM CaCl_2_, 140 mM K-gluconate, 0.01 mM EGTA, 4 mM Mg-ATP, 10 mM HEPES, osmolarity adjusted to 310 mOsm (with K-gluconate) and pH to 7.2 (with KOH). Signals were recorded at 50 kHz sampling rate, amplified, digitized and low passed-filtered at 5 kHz with patch-clamp amplifier (Double IPA Integrated Patch Clamp Amplifiers with Data Acquisition System, Sutter Instrument, USA). Spontaneous electrical activity of IO neurons were recorded in current clamp mode. For electrical stimulation experiments, we pulled <10 MΩ borosilicate capillaries filled with SPS and placed them dorsal to the border of the dorsal accessory olive (DAO), a region known to be traversed by bundles of glutamatergic excitatory afferents (Leznik et al., 2002). To evoke a transient STO phase-reset of IO neurons, we delivered 10 30 μs electrical pulses at 10 Hz (Stimulus isolation unit ISO-01D, NPI electronic, Germany). The stimulation intensity was adjusted starting from 30 μA and increased slowly up to 200 μA until stimulation artifacts were visible as fluctuations in the A6+ signal around the region of pipette insertion in brain tissue.

### 2.7. Bath Application of A6+ on Brainstem Slices

For bath-labeling of slices after post-slicing recovery period, they are transferred into a continuously-gassed 36°C SPS bath solution that includes A6+ in 3% DMSO for 30–480 s, taking special care to avoid mechanical movements that could damage the slices. After the designated incubation time, the slices are directly transferred into the recording chamber.

### 2.8. Assessing Slice Quality With “Patch-Score”

We designed “patch-scoring metric” to help in quantifying acute slice quality. While the calculation is somewhat subjective, we use it here to allow comparison of the degradation of slice health when exposed to DMSO for extended periods. The patch-score is built from a sum of 5 parameters (P1-P5), each one ranging between 5 and 1:

1. *Parameter 1: general visual features of the slice as seen in DIC imaging mode*. Score 5 = “neuron contours are clearly visible on DIC image, their diameter is around 20μm and neurons are abundant on the surface”; score 0 = “neurons are rare or not found at all, the slice is damaged, showing membrane aggregates and blebbing.”
2. *Parameter 2: general neuronal responses to poking with the patch-pipette:* Score 5 = “cytoplasmic membrane is resilient to light positive pressure from patch-clamp pipette and a small crater progressively forms and stabilizes around pipette tip”; score = 0 = “neurons explode after applying positive pressure on cytoplasmic membrane”; “neuron membrane is rigid and a crater doesn't appear on the pipette tip, sometimes neuron contour disappears.”
3. *Parameter 3: sealing behavior:* Score 5 = “the small crater disappears after releasing positive-pressure and the membrane progressively seals the pipette tip and a gigaohm resistance at the tip is reached within minutes”; score 0 = “neurons are sucked inside pipette after pressure release or the membrane breaks before sealing.”
4. *Parameter 4: whole-cell properties:* Score 5 = “input resistance of IO neurons is within 25–70 MOhm, the cell is hyperpolarized (oscillating IO neurons: −40 to −60 mV, non-oscillating IO neurons: −55 to −65 m), and the seal is stable for at least 10 min”; score 0 = “the input resistance is less than 10M Ohm or the cell is depolarized” (0/5).
5. *Parameter 5: IO-specific properties:* Score 5 = “If the neuron displays STOs, they are sinusoidal and oscillate in the 3–12 Hz range; if the neuron is spiking, a fast sodium component and an AHP can be clearly resolved and the frequency is lower than 1 Hz”; score 0 = “the oscillations are not smooth, membrane potential in unstable, or the cell is spiking faster than 1 Hz).”

### 2.9. Paramormaldehyde Fixation of Acute Slices After Bath-Application of A6+

After 45min of experiments, the slice is fixed in PBS containing 0.2% PFA at room temperature for 1 h, rinsed several times with PBS and immediately mounted on objective glass with Vectashield-DAPI (VectorLabs, CA) for immediate confocal imaging. The confocal imaging procedure and illumination parameters are the same as used for the anatomical study (see above). A6+ staining-score is determined from the A6+ to DAPI staining ratio within IO tissue.

### 2.10. Voltage Imaging Data Processing

Voltage imaging data were analyzed with Python 3.6. To analyze time-series images from IO slices stained with A6+ we use the following 3-step pipeline:

1. *Pre-processing*: image series were imported, cropped, corrected for bleaching and normalized for intensity histograms with Python Imaging Library (https://pillow.readthedocs.io/), scikit-learn (https://scikit-learn.org/) and scikit-image (https://scikit-image.org/) libraries.
2. *Frequency decomposition*: Three to twelve hertz power spectrum density (PSD) was computed for each pixel in the image with Welch method using *scipy* plugin (scipy.org). Frequency bands (3–12 Hz for STO) and absolute intensity Z-score were used to isolate signals of interest. A PSD value was obtained for each pixel and used to create a color-coded PSD-image normalized to the pixel that presents a maximal PSD for this frequency band. Then, the image was thresholded to 90% quantile for noise elimination.
3. *Clustering* Clusters of synchronously oscillating pixels were identified based on the thresholded PSD image using OPTICS (scikit-learn.CLUSTER.OPTICs, Ankerst et al., 1999; Schubert and Gertz, 2018) method for unsupervised clustering. The algorithm runs using a minimal number of observations per cluster that we set as the minimal number of pixels containing one IO neuron. To calculate the minimal number of pixels to fill one IO neuron oriented on a coronal slice, we used the short radius of the ellipse circling the dendritic arbor of 20 IO neuron 3D reconstructions available online (neuromorpho.org/) (Vrieler et al., 2019). The average diameter of the ellipse is 110 ± 12 μm. With 5x magnification, one pixel size is 18 × 18 μm^2^ and thus, one full IO neuron is contained around 29 pixels. We used a minimum cluster size of 5 pixels to ensure the cluster size includes a significant portion of the surface of one IO neuron in coronal orientation.

Detailed python code for analysis is available online in repository. https://github.com/oist/Frontiers_ANNINE-6plus_IO_STO

### 2.11. Electrophysiology Data Analysis

For cross-correlation analysis between imaging and electrophysiological recording, 50 kHz current-clamp traces of STO were first down-sampled to the frequency of acquisition of voltage images (60–120 fps). Time-lagged cross-correlation is calculated with python using *numpy, scipy* and *pandas* plugins on 1 s epochs of signal.

### 2.12. Statistical Analysis

All data are given as mean ± SEM. Variance between individual observations of group data were tested with Levene test for variance equality between two groups. In case of equal variances, group data were tested with Student *t*-test or ANOVA as indicated. In case of unequal variance, Welch *t*-test was used. All statistics were calculated in Python using the Scipy plugin (scipy.org).

## 3. Results

### 3.1. Mesoscale Spread of ANNINE-6plus During Post-operative Labeling Period

As an amphiphilic dye, A6+ will immediately and indiscriminately label any lipid membrane as soon as it comes in contact. In order to obtain voltage recordings from a wide area, the experimentation should be delayed until the dye has diffused to a suitably-broad area.

**Figure 1:**
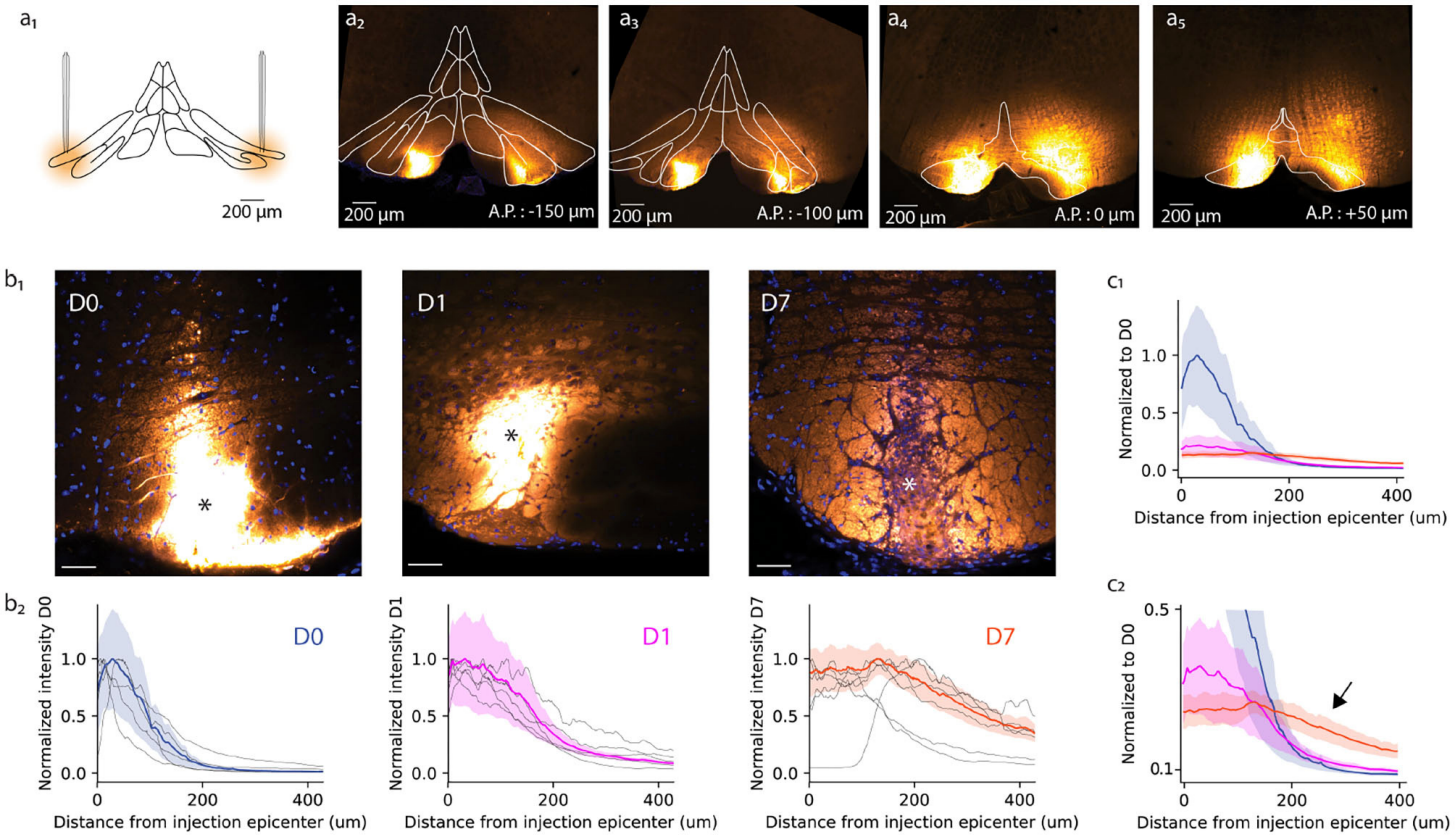
Anatomical study of A6+ spread within the inferior olive. **(a)** Schematic representation of bilateral injection of A6+ into dorsal accessory olive. **(a2–a5)** Example of A6+ spread in medio-lateral and antero-posterior directions imaged a few hours after injection. **(b1)** Mesoscale A6+ staining intensity becomes more uniform after injection (D0, D1, and D7 signify time points of a few hours, 1 and 7 days, respectively). **(b2)** Quantification of the changes shown in **(b1)**, represented in a radial plot profile as a peak intensity ratio. **(c1)** Radial plot profile normalized to D0 average peak intensity value shows that A6+ is cleared quickly from the injection site between D0 and D1, and further but slower until D7. **(c2)** This decrease is accompanied by an broadening of the stained zone (indicated by arrow).

To establish the optimal incubation period for A6+ staining, we quantified the extent, uniformity and cellular homogeneity of A6+ fluorescence following dye injection into the IO (lateral principal and dorsal accessory olives; Figure 1a1) at three different time points: 1 h after the injection [Day (D)0], on the next day (D1), and a week later (D7). Three mice were perfused at each time point, and confocal image stacks were obtained from the stained tissue (example images from a single animal are shown in Figures 1a2–a4). As mentioned in the section 2, A6+ injected into the left and right IO was dissolved in 1 or 3% DMSO. We did not observe an obvious difference between these two conditions (data not shown), and, therefore, pooled the data.

First, we quantified the effect of a longer waiting period after dye injection on the mesoscale spread of A6+ within the IO. For this purpose, radial fluorescence intensity profiles centered around the injection center (indicated by asterisk in Figure 1b1 for the representative example slices obtained at D0, D1, and D7) were constructed (see section 2). Comparing the distribution of intensity profiles among the three different incubation periods (Figure 1b2) revealed that the strongly labeled region observed immediately after dye injection (D0; Figures 1b1,b2) has a radius of 84 ± 15 μm from the injection center (half-maximal intensity, *n* = 6 slices from 3 animals). The intensity profile, showing the expected diffusion profile, was significantly broadened in animals perfused 1 day later (half-maximal intensity range 153 ± 17 μm, *p* < 0.05; Figures 1b1,b2, middle panels) and even more so 1 week after dye injection (half-maximal intensity range 388 ± 48 μm, *p* < 0.05). The peak staining intensity drops around 60% between D0 and D1 but does not decrease further until D7 (Figure 1c1). Also, the A6+ intensity profile looses its bell shape and acquires a prominent “tail” reaching hundreds of micrometers from the injection center (Figure 1c2; black arrow).

### 3.2. ANNINE-6plus Internalization in Neurons

**Figure 2:**
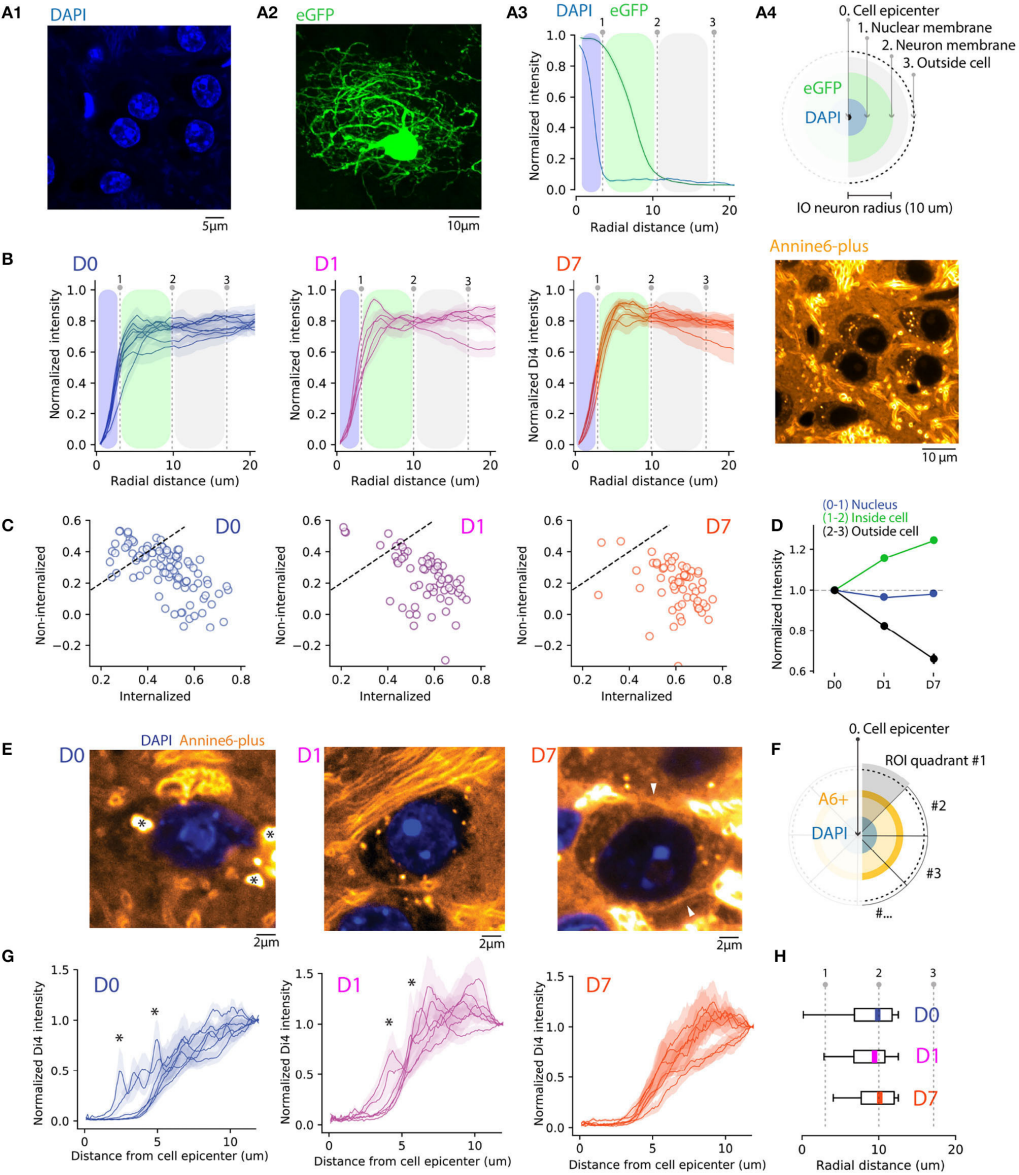
Quantifying A6+ internalization by IO neurons. **(A)** Defining subcellular boundaries of IO neuron somata using cytosolic (eGFP) and nuclear (DAPI) labeling. Panel **(A1)** shows example of eGFP. **(A1)** eGFP labeled IO neuron as well as IO neuron nuclei labeled with DAPI in **(A2)**. Panel **(A3)** depicts methods for calculating radial profile plot of the two stainings as well as their intensity profiles. The blue shaded region is an estimation of IO nucleus coverage (dotted line indicating the border) while the green shaded region depicts extent of cell soma. **(B)** Progressive internalization of A6+ from **(D0–D7)** calculated from 20x confocal images. Each line is an average (MEAN ± SD normalized to the maximal value of the distribution) of 30 cells from 6,5,5 slices in **(D0,D1,D7)**, respectively. Dotted lines represent the borders of nuclei and cell bodies represented in **(A)**. The emergence of a peak between the 2 lines (1-2) shows a progressive shift of the intracellular-neuropil labeling ratio toward intracellular of A6+ with time. **(C)** Average value of staining intensity for individual neurons [included in **(B)**] in the cytosol (1-2) and neuropil (2-3) showing a progressive shift toward cytosolic labeling. **(D)** A6+ intensity change with time is shown for the 3 compartments as normalized to D0. **(E)** Example of magnified IO neurons (40x confocal image) showing improvement of A6+ staining quality over time. The cell membrane is visible in D7 (white arrowheads). The strong fluorescence visible in the extracellular space in the example image for D7 originates in the myelin sheath of passing axon bundles. **(F)** Diagram illustrating the process of radial profile plot segmentation on A6+ confocal images quantified in **(G)**. On data normalized to background where the variability of peak intensity is representative of membrane staining within individual neurons (D0,D1,D7, *n* = 7 neurons, MEAN ± SEM). **(H)** Boxplots (MEDIAN ± SD with quantiles) showing the reduction of peak intensity variability.

To gain further insights into the changes in A6+ distribution in the post-injection period, we compared the staining of neuronal somata with that of the neuropil. For this purpose, we first obtained the distribution of DAPI-stained IO neurons' nuclear radii using high-magnification (40x) confocal images (*n* = 227 neurons; Figure 2A1), as well as a distribution of soma sizes from IO neurons sparsely labeled with GFP (*n* = 50 neurons from 3 animals; Figure 2A2 and Supplementary Figure 1). Using these measurements (shown in Figure 2A3) with conservative threshold values at 0.25 quartile as limits led us to define radial distance limits for IO neurons' nuclei and somata (schematically depicted in Figure 2A4; nuclei 0-3.5 μm; cytosol, 3.5–10 μm; neuropil, >10 μm).

Next, we quantified the A6+ staining among nuclei, somata without the nuclei, and neuropil using confocal images of A6+ labeled neurons (226 neurons in 10 slices and 5 animals) located within 300 μm radius from the injection center. Here, we computed average 360-degree radial intensity profiles of A6+ stained neurons, normalized between the minimum and peak intensity in each animal (see section 2 for details; Figure 2B). We found that at D0, regions outside the soma were a major contributor to the gross fluorescence around neurons in a given slice (peak intensities found at distances 18.6 ± 2.1 μm from cell center; *n* = 99 neurons, 3 animals at D0). This ratio between neuropil and cytosol localization shifts toward the cytosol on D1 (peak distance at 16 ± 3.1 μm from soma center; *t* = 0.81, *p* = 0.43 compared to D0), and even more on D7 (peak intensities found at 8.21 ± 1.6 μm; *t* = 4, *p* = 0.002 compared to D0) demonstrating that on population level, the fraction of dye that is internalized increases over incubation time (One-way ANOVA; D0-D7; *f* = 13.6, *p* < 0.01). The same trend is evident when comparing the relative amount of fluorescence observed in internal vs. external compartments (Figure 2C). At D0 and D1, 55 and 31% of neurons have more than 50% of the signal originating in the intracellular domain, while at D7 this fraction increases to about 87%.

We conclude that concomitantly with the diffusive spread of A6+ in brain tissue and clearing of A6+ from the extracellular leaflet of the cytoplasma membrane during post-injection days, A6+ is increasingly accumulating and retained intracellularly, so that at D7 the relative contribution to the fluorescence signal from intra- vs. extrasomatic compartments increases as visualized in Figure 2D (D1 +15.7 ± 3%, D7 +24.5 ± 3.3% signal strength increase relative to D0; neuropil signal strength decreases 17.5 ± 7.1%, and 33.9 ± 7% at D1 and F7, respectively). This leads to an overall increase in the cytosol-to-neuropil fluorescence ratio of almost 60%. Notably, the absence of A6+ staining inside nuclei suggests that A6+ -labeled neurons remain intact at least 1 week after injection (−3 and −1.5% decrease of nucleus staining for D1 (*f* = 0.22, *p* = 0.63; ns) and D7 (*f* = 0.048, *p* = 0.8; ns), respectively, compared to D0.

### 3.3. Longer Staining Time Improves Homogeneity of ANNINE-6plus Staining on Single-Cell Level

To assess the uniformity of somatic labeling, specifically related to relative contribution of fluorescence signals originating from the neuronal membrane vs. internal organelles, we constructed radial intensity profiles for 20-degree segments for 6 IO cells for D0, D1, and D7 (Figure 2E). Normalizing the intensities to the values measured at the periphery of the region of interest and examine the averaged profiles (Figure 2G). In line with the analysis shown in Figure 2B, the profiles show that the peak A6+ fluorescence is located outside of the cell body at D0 and progressively becomes more narrowly distributed around the region corresponding to the cytosol. Also, the neuronal membrane becomes visible in many neurons by D7 (Figure 2E, right panel, white arrowhead), suggesting progressive accumulation in the membrane. On D0 and D1, numerous spherical fluorescent puncta can be seen (0.2–1 μm in diameter; indicated in Figure 2E with asterisks). These puncta become less prominent over time as the staining becomes increasingly uniform, and at D7 they were only rarely observed, as can be also seen in the smoothening of averaged segmented radial profile plots (Figures 2F–H; ks-test: *k* = 0.4, *p* < 0.001 between average intensity distributions for D0 and D1 and *k* = 0.191, *p* = < 0.05 between D0 and D7). Taken together, these results indicate that a 1-week-long *in vivo* waiting period after dye injection results in improved homogeneity of labeling. It is possible that this results from slow dissolution of dye aggregates trapped within the somata, that eventually will label the cytosolic leaflet of the plasma membrane or intracellular membranes. In contrast, dye dissolved from aggregates in contact with the extracellular space gets washed away.

### 3.4. Longer Labeling Period Enhances Signal-to-Noise Ratio of Fluorescent Voltage Imaging

Having established that the A6+ labeling is more uniform 7 days after injection than immediately after, we proceeded to investigate whether this improvement in staining correlates with the quality of signals obtained from inferior olive neurons expressing subthreshold oscillations (STOs).

**Figure 3:**
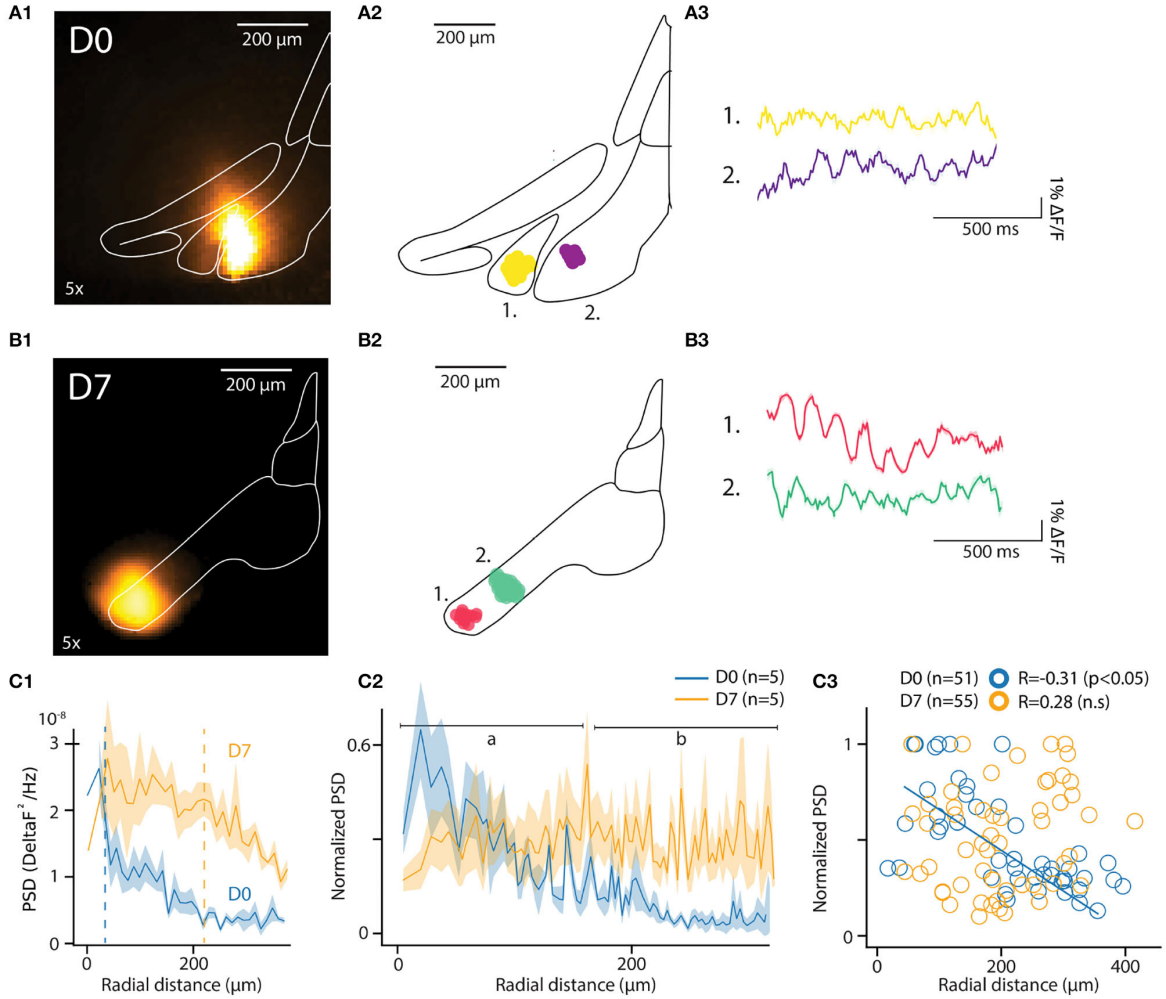
*In-vitro* optical recording of IO-STO with A6+. **(A1,B1)** Maximum projection image of an example recording from mice injected a few hours (D0) or 1 week (D7) before *in-vitro* experiments (left images), the injection spot appears in orange inside the delimited IO. For each condition (D0, D7) the central diagram shows 2 clusters of synchronously oscillating pixels with their respective average traces on the right. **(A2–B2)** PSD of pixels in function of their distances from the injection center for both D0 and D7 conditions. d1. The average 4–12 Hz PSD value of each pixel cluster (normalized to the strongest cluster from each slice, *n* = 5 slices per condition) is represented in function of the distance between their cluster centroid from the injection center. Note the negative correlation in D0 compared to the absence of correlation in D7, with a population of strongly oscillating clusters 300 μm away from soma (black arrow). **(C1–C3)** Decrease of the 4–12 Hz PSD of individual pixels for D0 but not for D7 with the distance from injection center **(C1)** and representation of average values **(C2)** for proximal [**(A)** 0–150 μm] and distal [**(B)** 150–300 μm] compartments around center. In **(C3)**, individual STO-PSD datapoints for clusters of pixels around injection center in both D0 and D7 conditions showing anti-correlation for D0 but not for D7.

To this end, we acquired voltage image series with a wide field of view (5x objective field of view 3 × 3 mm; pixel size 17.86 × 17.86 μm^2^) encompassing the entire IO (examples of maximum projection images shown in Figures 3B1,C1, for D0 and D7, respectively). Based on reported IO cell density (Devor and Yarom, 2002a), a single pixel with this magnification is the size of 1.07 ± 0.7 IO cell somata. Thus, fluorescence signals from individual pixels necessarily represent summation of voltage changes in several neurons. Establishing the actual number of neurons contributing to the signal is beyond the scope of this paper but depends strongly on the resolving power of the optical system used.

In order to reveal the spatiotemporal organization of ongoing voltage fluctuations and investigate whether stable oscillatory signals matching STO properties can be recorded with this method, we used unsupervised clustering (see section 2) to delineate pixel groups with shared voltage fluctuation signatures in the 3–12 Hz band.

In each slice, the algorithm identified multiple clusters as aggregates of pixels within the IO boundaries. The location of 2 of them, each approximately 100 μm in diameter, is shown for representative example slices in Figures 3A2,B2 (D0 and D7, respectively). The number of clusters and their distances from injection center were comparable at D0 and D7 [6.4 ± 1.5 and 5 ± 0.8 clusters in *n* = 5 slices at D0 and D7; *t* = 0.8, *p* = 0.4; distances from injection 135.4 ± 10.3 μm and 113.6 ± 10 μm for D0 and D7, (*t* = 1.13, *p* = 0.1)]. Cluster sizes were similar between D0 and D7 samples (8.3 ± 0.4 and 8 ± 0.5 pixels, corresponding to 20507 square μm and 21868 square μm areas for D0 and D7, respectively; *t* = 0.43, *p* = 0.66). These characteristics are in line with previously-reported reconstructions of IO cells (Rekling et al., 2012; Kølvraa et al., 2014; Vrieler et al., 2019).

Average fluorescence signals from all pixels in the clusters express clear oscillatory activity in the 3-12 Hz band. Figures 3A3,B3 show traces obtained from the example clusters depicted in Figures 3A2,B2). The pixels forming a cluster were located within a range of 135.4 ± 10.3 μm for D0 and 113.6 ± 10 μm for D7 (*t* = 1.13, *p* = 0.1) from the center of a cluster (for D0 and D7, respectively). Notably, the pixels did not necessarily cover a continuous area, suggesting that a coherent activity between distinct, tightly-coupled IO clusters may be promoted by sparser, long-range bridging connections.

The fact that oscillations matching expected IO STO features could be recorded without averaging from clusters in both D0 and D7 samples was encouraging. To further investigate whether the observed variation in oscillation amplitude was linked to labeling strength, we compared the spatial distribution of power spectral density of the recorded fluorescent signals between D0 and D7. As shown for the data from all pixels within example slices in Figure 3C1, the peak PSD in the STO band was similar in D0 and D7. However, the PSD drops rapidly with distance from the injection center at D0 while at D7 it stays relatively constant across larger distances (half-maximal PSDs at 34 and 218 μm for D0 and D7, respectively; indicated with dashed lines in Figure 3D1). When the same analysis was performed on pooled clusters identified in all slices at D0 and D7 (Figure 3C2; 5 slices from 2 and 3 animals for D0 and D7, respectively), it became clear that at D0, the peak PSD drops rapidly within tens of μm from the injection site while at D7, the voltage fluctuations can be observed with uniform power across hundreds of μm. This observation is corroborated by the negative correlation between distance from injection center and PSD (normalized to maximal PSD from individual recordings) at D0 (*R* = −0.35, *p* < 0.01) but not at D7 (*R* = 0.24, *p* = 0.07; Figure 3C3).

Taken together, the power spectrum analysis of A6+ fluorescence fluctuations is in line with our anatomical observation that waiting 7 days post-injection improves the SNR of voltage signals recorded in the inferior olive. Importantly, the extended dye labeling period did not decrease the total number of clusters identified or the PSD (*t* = −0.856, *p* = 0.395) and STO-PSD distributions were similar at D0 and D7 (ks = 0.127, *p* = 0.77; data not shown). This, combined with similarities in number and count of identified clusters, suggests that an extended labeling period does not affect IO circuit structure in a way that would have a detrimental effect on IO network function.

### 3.5. Wide-Field Responses in IO STOs to Electrical Stimulation of Excitatory Afferents

In earlier studies, a voltage imaging approach has been used to examine network-wide IO STO patterns in response to electrical stimulation of excitatory afferents (Llinás et al., 2002; Leznik and Llinás, 2005). In those works, stimulation-triggered averaging of voltage images revealed the emergence of distinct, temporary oscillating clusters. While instrumental to understanding of the mechanisms driving clustered STO activity, trial averaging prohibits observing subtle differences among trials. We wondered if such stimulation-evoked cluster emergence could be observed in single-trial recordings without averaging with the present, improved imaging capability.

**Figure 4:**
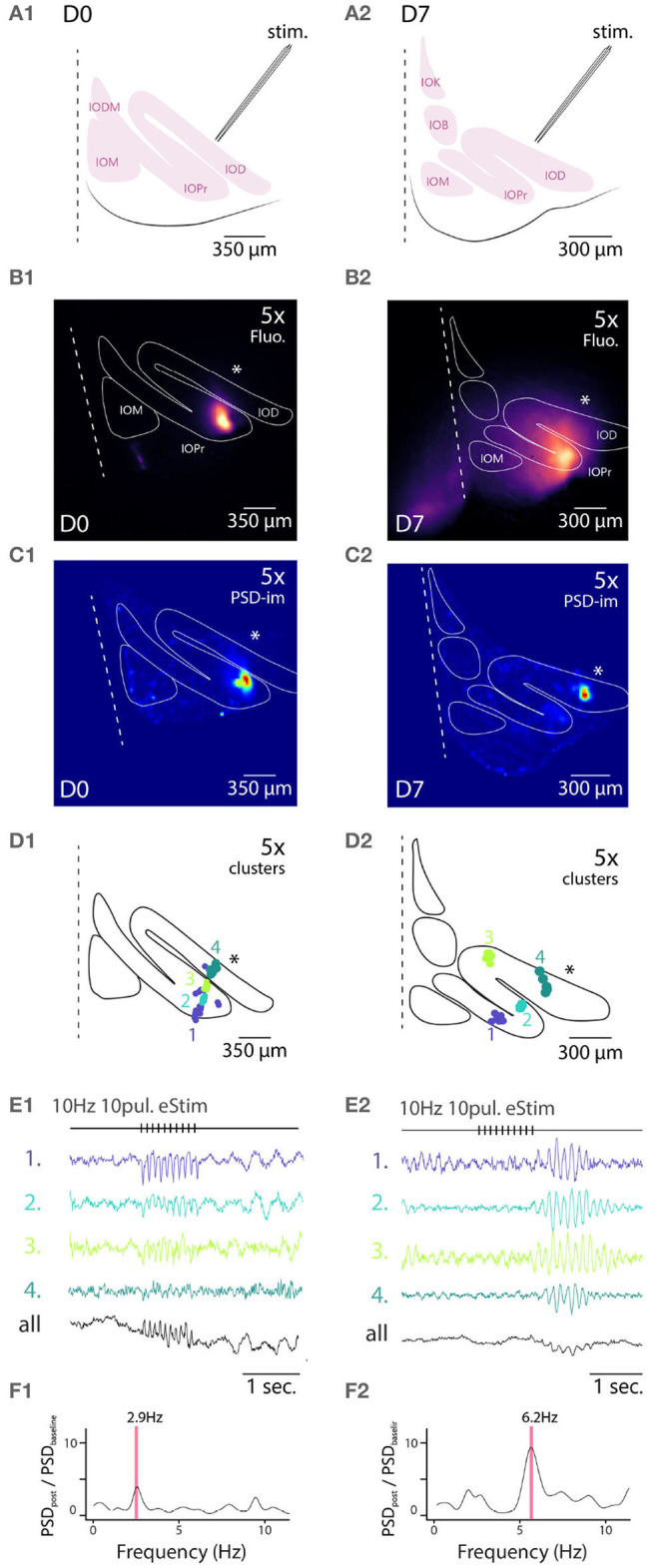
Wide field 1-photon voltage imaging of STO in acute brainstem slices. Example of one experiment in D0 condition **(A1–F1)** and in D7 condition **(A2–F2)**. Glutamatergic afferent fibers are electrically stimulated as shown in the representation panels **(A1,A2)**. Panels **(B1,B2)** show the maximum projection image of a 10 s recording made *in-vitro* in D0 and D7 condition, respectively. White lines delineate the borders of IO sub-nuclei and stimulation pipette is marked by asterisk. Image pixels are processed individually to get PSD values for the 3–12 Hz frequency band: panels **(C1,C2)** are PSD-images color-coding the strength of 3–12 Hz oscillations. Panels **(D1,D2)** show the spatial position of 4 color-coded clusters in each each condition and their associated traces in **(E1,E2)**. Panels **(F1,F2)** display the PSD spectrum of evoked STO rebound after stimulation normalized to PSD spectrum 2 s before stimulation. The red band marks the frequency of STO evoked by electrical stimulus.

To this end, we used slices obtained from animal from either D0 or D7 staining conditions. To isolate excitatory afferent input to the IO network, 100 μM of picrotoxin was added to the bath to block GABA_*A*_-receptor-mediated synaptic input, and a stimulation electrode was placed outside of the IO to activate afferent glutamatergic axons (see section 2 for details; arrangement schematically depicted in Figures 4A1,A2). Figures 4B1,B2 show maximum projection of wide-field fluorescence images of the representative example slices.

In all slices examined (*N* = 5 and 5 for D0 and D7, respectively), delivery of 10 pulses at 10 Hz resulted in emergence of clear oscillating clusters visible across the entire IO network as evident in the spatial distribution of 3–12 Hz PSD (Figures 4C1,C2). As was described above, we identified pixel groups of synchronously oscillating intensities (example clusters indicated with different colors in Figures 4D1,D2) and examined the averaged detrended data (from 0.25 s window median filtered trace subtraction) fluorescence traces obtained for each cluster (examples for the clusters depicted are shown in Figures 4E1,E2). During the stimulation train, fast negative deflections were evident in the average fluorescence signals from these cluster (Figures 4E1,E2). While we did not investigate further the waveform of these fast responses, it seems they reflect a combination of afferent signals and postsynaptic responses. Importantly and in line with previous results (Llinás et al., 2002; Leznik and Llinás, 2005), the stimulation invariably resulted in enhancement of oscillations at least in some clusters in all *n* = 5 slices at both D0 and D7, as evidenced by the power increase at specific frequencies when comparing recordings before and after stimulation (Figures 4F1,F2).

Similarly to the results obtained from comparing oscillation PSDs between D0 and D7 (Figure 3), we observed that the stimulation-evoked STOs were stronger in samples obtained after 7 days of labeling. While STOs were clearly discernible already at D0, albeit with lower signal-to-noise ratio, they were seen across wider regions of the IO at D7 (with an average of 166 μm and a maximal 366 μm distance between 2 clusters for D7).

Taken together, the STO-PSD signals can be used to reveal both spontaneous and stimulation-evoked STOs across the entire IO network.

### 3.6. ANNINE-6plus Fluorescence Reliably Reports STOs on Single Cell Level

**Figure 5:**
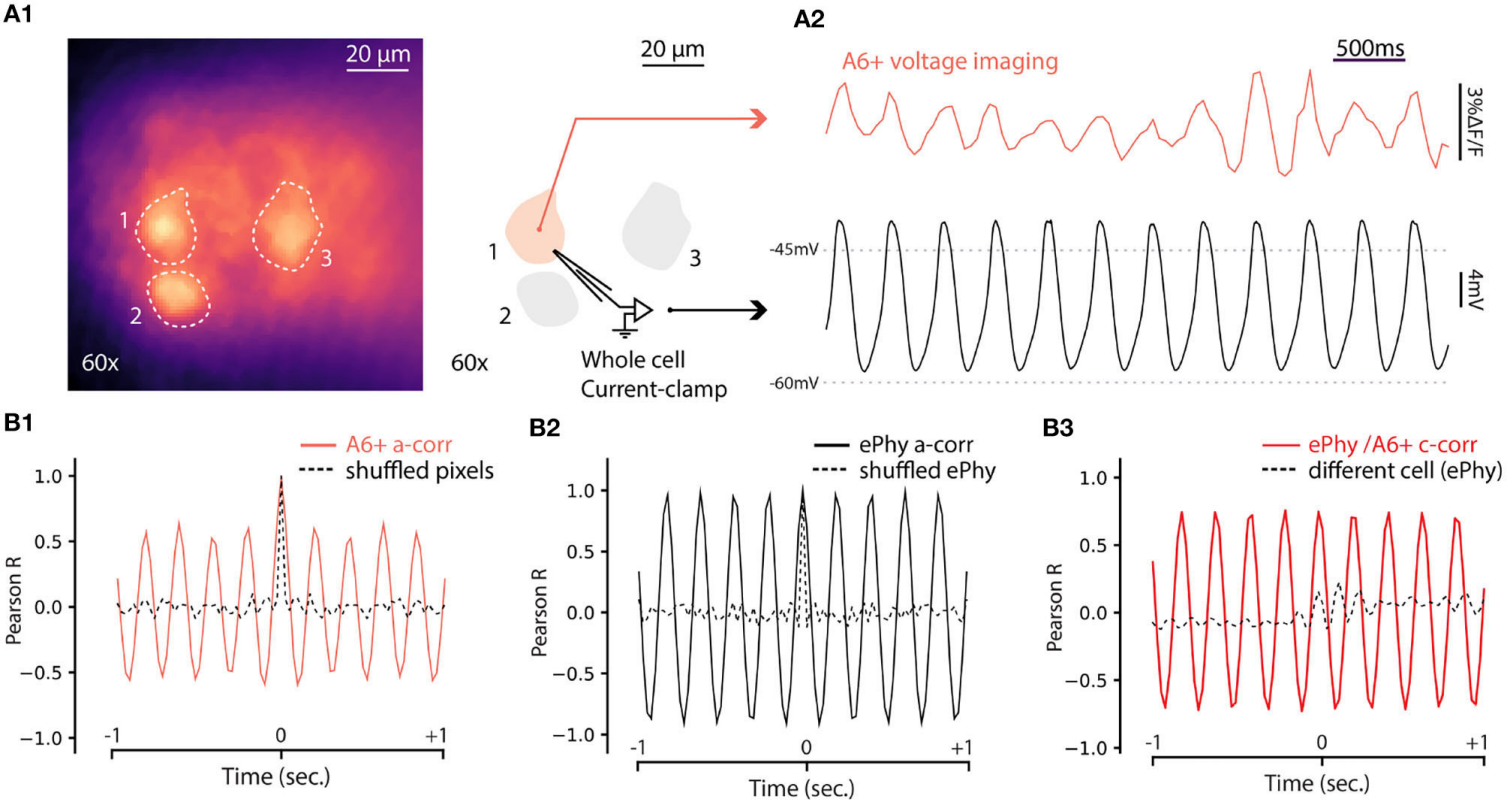
Simultaneous voltage imaging with A6+ and whole cell current-clamp recordings of STO. **(A1)** Maximum projection image of a 10sec recording of IO neurons stained by A6+ 7 days after injection. On the right, schematics of experiment where one of the stained neurons is recorded in whole cell current clamp configuration. **(A2)** Top trace (purple) is the average of pixels from a1 in function of time. Bottom trace (black) is the membrane voltage simultaneously recorded from the same neuron. **(A3)** One second window sliding auto-correlogram of voltage imaging trace (purple trace) and equivalent with shuffled pixels (dashed line). **(A4)** One second window sliding auto-correlogram of membrane voltage and shuffled trace (dashed line). **(A3)** One second window sliding cross correlogram between A6+ imaging recording and membrane potential recording (red trace). Similar analysis was performed with un-paired recordings where electrophysiological recording of STO was taken from another cell (black dashed trace).

The results presented above establish that fluorescence signal from A6+ can be used to reveal coherent oscillatory signals originating in groups of IO neurons in single trials. While this is a significant improvement to the previous reports (Devor and Yarom, 2002b; Leznik et al., 2002), lack of true single-cell resolution prevents investigation of mechanisms generating the network oscillations. To explore the capabilities of A6+ imaging with respect to reporting oscillations on single-cell level, we combined fluorescence imaging under 60x objective with whole-cell patch-clamp recordings in current clamp mode. As shown in Figure 5A1, A6+ -labeled IO cell somata were clearly distinguishable in slices, allowing targeted patching and comparison of electrical and optical voltage measurements in the same cell. Indeed, in all cells that expressed STOs in the electrical recording of membrane voltage (trace from the example cell indicated in Figure 5A1 is shown in Figure 5A2, black trace), clear fluctuations were seen in A6+ signals averaged from the areas encompassing the patch-clamped somata (Figure 5A2, red trace). The optical voltage signal correlates with the electrical signal which indicates that the A6+ molecules are located on the intracellular leaflet of the plasma membrane (Kuhn and Roome, 2019). Even though the waveform of oscillation as recorded in fluorescence signal was somewhat less stable than in intracellular voltage recording, 1-s 3sliding autocorrelogram of the fluorescence signal revealed a sinusoidal oscillation at 4.88 Hz, 18 times stronger than shuffled signal (Figure 5B1; autocorrelation peaks for A6+ signal on this example cell: 0.603 ± 0.05; shuffled: 0.03 ± 0.01). This shows that voltage signals recorded from IO neurons with A6+ probe display matching characteristics of the STOs as observed in intracellular current-clamp recordings in the same neuron (auto-correlation on V_*m*_ trace: 4.87Hz with peaks: 0.97 ± 0.006; shuffled: 0.02 ± 0.006; Figure 5B2) despite possible signal contamination from fluorescence originating in intracellular membranes. The A6+ and V_*m*_ signals were tightly correlated (c-corr peaks: 0.74 ± 0.04; Figure 5B3, red trace; compare with lack of correlation with unpaired V_*m*_ recording, black trace) demonstrating that A6+ fluorescence signal can be reliably used to investigate the frequency and phase features of IO STOs (average c-corr peaks for *n* = 5 cells: *R* = 0.4 ± 0.09; shuffled: 0.01 ± 0.04 *p* < 0.01; unpaired bootstrap: 0.2 ± 0.05 *p* = 0.1).

### 3.7. STO Responses to Electrical Stimulation in Sub-cellular Compartments

**Figure 6:**
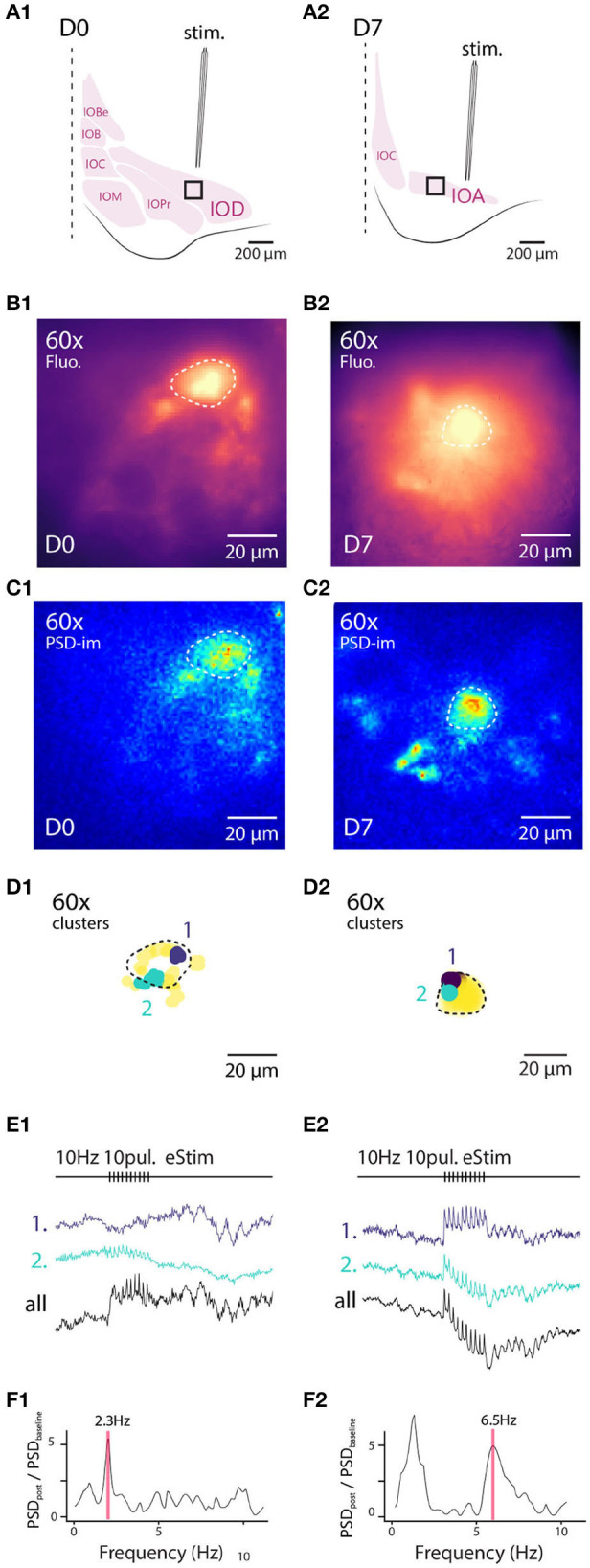
High-resolution 1-photon voltage imaging of STO. Example of 2 IO neurons and their close environment from D0 **(A1–F1)** and D7 **(A2–F2)** imaged at high magnification with 120 Hz sampling rate (120 Hz). Top panels are schematics of experimental setup for both cells (similar as Figure 5). Panels **(B1,B2)** are maximum projection images of 10 s recordings where the cell soma are distinguishable from background and circled with dashed white lines. PSD density is calculated for each pixel and represented on panels **(C1,C2)**. Two clusters of oscillating pixels are calculated and represented in **(D1,D2)** in distinct colors. Cell soma is delineated with a black dashed line. Cluster are following different time-courses during and following electrical stimulus **(E1,E2)**. The average PSD of all the oscillating pixels **(F1,F2)** shows preferred STO frequency following electrical stimulation as well as frequency shift on the right panel.

Next, we examined how electrical stimulation modulates STOs on the level of single neurons with the same experimental configuration as in Figure 4 (Figures 6A1,A2). For this purpose we selected neurons that showed a clearly-labeled neuronal membrane, suggesting high level of membrane integration of A6+ (Figures 6B1,B2) and recorded fluorescence fluctuations before and after a train of electrical stimuli delivered as described above in slices from both D0 and D7.

Again, we identified clusters of pixels expressing synchronous fluorescence fluctuations in the 3–12 Hz frequency range as was done with the wide-field data (PSD density images shown in Figures 6C1,C2, and identified example clusters are indicated as purple and green spots in Figures 6D1,D2). The clusters were mainly found near the outer boundaries of IO somata, as evidenced by the fact that the average pairwise Euclidean distances between pixels (17.5 ± 0.75 μm, *n* = 8 neurons) closely matched the average soma diameter (Figure 2A), in line with the oscillatory signal originating from neuronal membrane rather than intracellular organelles. Representative traces obtained from D0 and D7 samples are shown in panels 6e1 and e2 for averaged signals from two identified oscillating clusters (indicated with colors) as well as from the average over soma area (black line), demonstrating that the somatic STO could be recorded from areas much smaller than the soma itself when A6+ labeling is strong (such as in D7 sample, Figure 6B4). Furthermore, the fact that the signal obtained from whole-soma regions shows oscillatory behavior with an amplitude comparable to individual clusters (compare cyan and black traces in Figure 6E2) suggests that fluorescence originating from intracellular organelles does not seem to greatly confound STO monitoring. In all cases examined (*n* = 8 cells), the baseline STO (peak frequency 4.7 ± 0.75 Hz) increased in power above baseline z-score (5.7 ± 2-fold) immediately after the stimulation (traces from example clusters shown in Figures 6E1,E2; comparison of oscillation power before and after stimulation in Figures 6F1,F2). Furthermore, after the stimulation the oscillation sometimes seemed to obtain a new frequency component (compare oscillation shape before and after stimulation in Figure 6E2).

### 3.8. Electrical Stimulation Shapes the Spatiotemporal Organization of IO STOs

**Figure 7:**
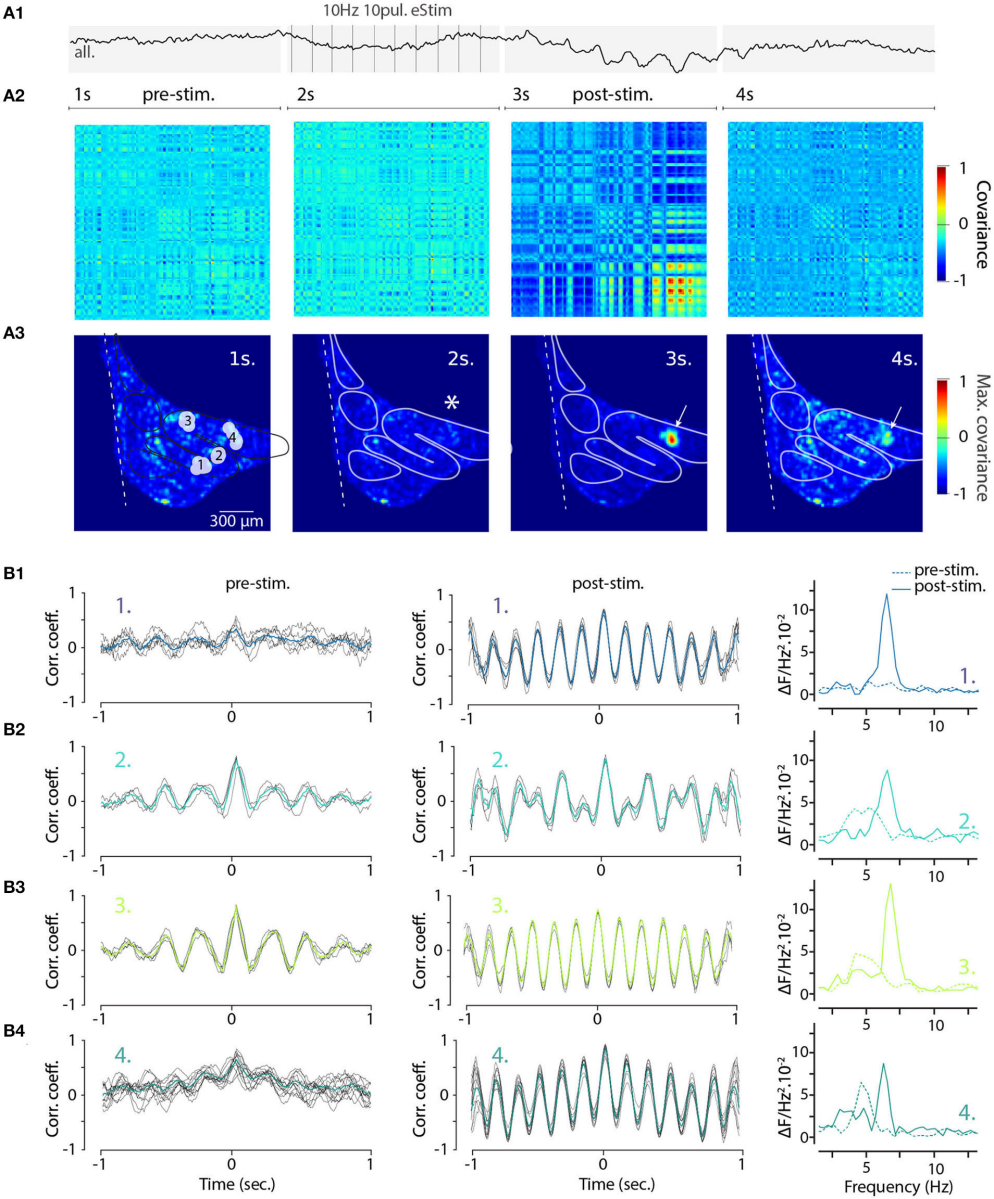
Increased coherence and frequency-shift of STO from wide-field recorded IO neurons after electrical stimulation. **(A1)** Average 4 seconds trace of all 140 oscillating pixels detected in one recording of electrically stimulated IO (clusters and traces also shown in Figures 4D2,E2). Panel **(A2)** shows the corresponding covariance matrix (color-code normalized to maximal and minimal covariance) between all 140 pixels for each of the 4 periods of the recording, before (1–2 s; pre-stim), during, and after electrical stimulus (3–4 s; post-stim). Coherence image **(A3)** is calculated from maximal covariance of matrices in **(A2)**. White arrow indicates a local increase of pixel covariance after stimulus. On **(B)**, the 2 s sliding cross-correlograms for 4 clusters of pixels (see Figures 4D2,E2) comparing cluster coherence before and after stimulation using correlation coefficient (left and central panels). Right panels show the frequency of STO before (dashed lines) and after (plain lines) stimulus for each of the clusters.

To gain further insight into how electrical stimulation shapes the frequency and coherence of IO STOs and as a proof-of-concept example of the utility of single-trial measurement of network-scale subthreshold activity, we returned to the wide-field view of the IO. Here, using the voltage recording shown in Figure 4 (right panels) we selected 140 oscillating pixels within the IO structure from STO-PSD image. As is shown in their averaged fluorescence signal trace (Figure 7A1), stimulation results in transient emergence of a clear and global oscillations with main frequency around 6 Hz. Intriguingly, even though this analysis only contained data from pixels that oscillated during the trial, the prominence of the stimulation-evoked network-wide oscillation was less impressive than what was seen in individual clusters (Figure 6E2).

There are two interesting possibilities for interpreting this observation. First, it could be that the stimulation-evoked oscillation frequencies differ between clusters and the average signal would thus have lower amplitude. Second, even though the evoked oscillations within clusters would have the same frequency, there would be a non-zero phase-shift between the clusters.

To visualize the stimulation-evoked changes in the oscillatory landscape, we composed cross-covariance plots of the 140 pixels in the 4-s trial data described in Figure 4. The voltage image series were divided into four time windows corresponding to baseline, stimulation, post-stimulation and recovery periods (Figure 7A2; panels correspond to the timeline of the trace shown in Figure 7A1, and the spatial arrangement of covariance maxima are shown in Figure 7A3; stimulation location is marked by asterisk in second panel). Somewhat surprisingly, the electrical stimulation itself did not immediately increase in overall synchronicity of the IO (Figures 7A2,A3, second panels); however, the cessation of the stimulation was followed by the emergence of regions with strong coherence (red areas in Figures 7A2,A3, third panels). The increase was most pronounced near the stimulation area (arrow in third panel, Figure 7A3) and this signature of the enhanced IO-wide coherence is maintained for several seconds after the stimulation ends, suggesting a long-lasting modification of the network state.

**Figure 8:**
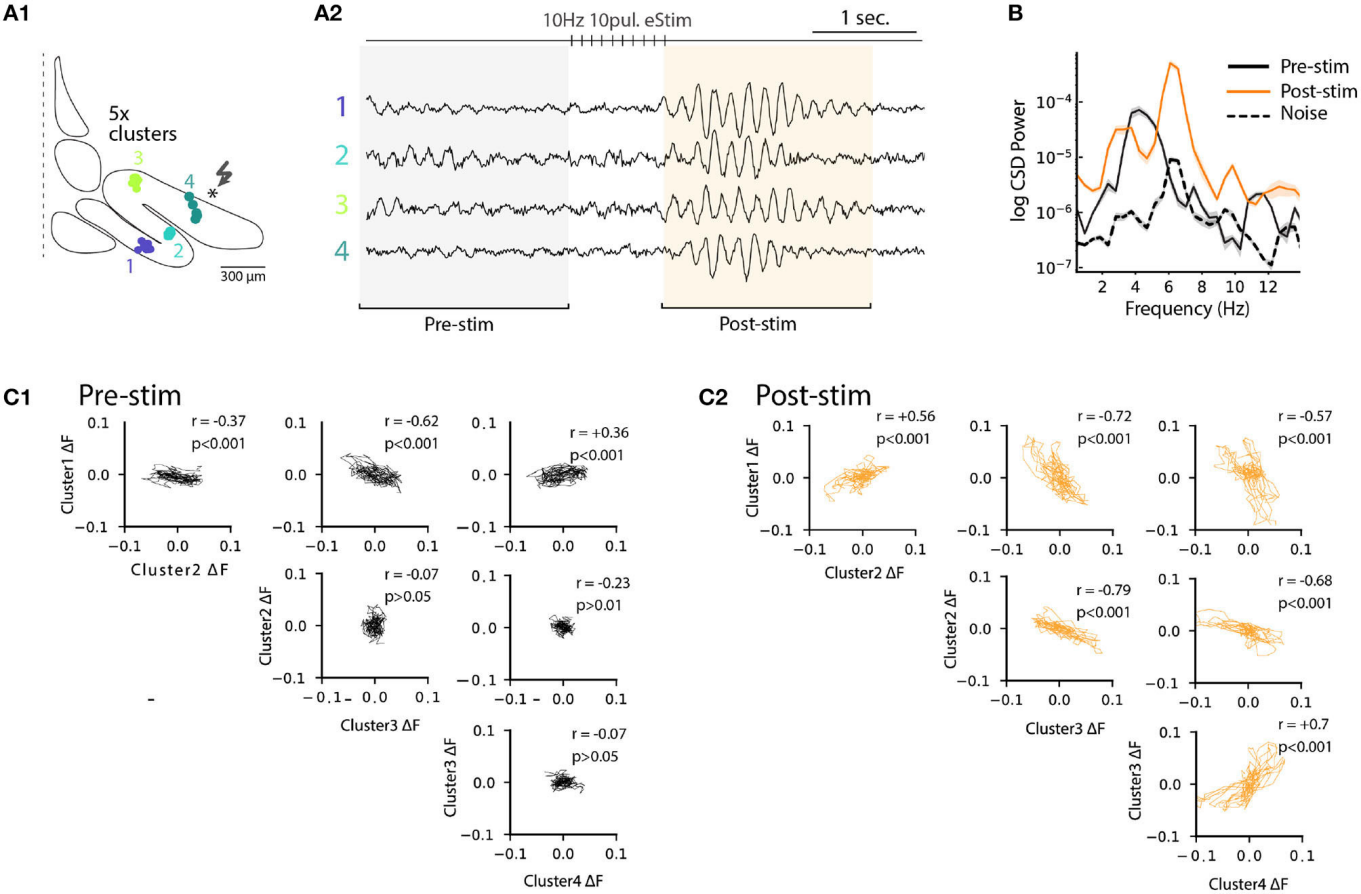
Phase-shift of STO after excitatory input stimulation. **(A1)** Diagram representing 4 cluster positions on inferior olive slice (see also Figure 4). **(A2)** Average voltage imaging traces from the 4 corresponding clusters. **(B)** Cross-power spectral density (average CSD, ±SEM) of the 4 clusters before (black trace) and after (orange trace) electrical stimulation. Average CSD between cluster traces and noise is represented with dashed trace. **(C1)** Time-point correlation matrix between average traces of the 4 clusters before stimulation with the corresponding correlation coefficient inside each subplot. **(C2)** Correlation matrix as in **(C1)** after electrical stimulation.

Previously (Figure 6), we noted that electrical stimulation can lead to frequency shifts in single oscillating neurons. To explore the generality of this observation, we constructed cross-correlation graphs for 4 clusters identified in the image sequence (Figure 7B). Even though they display different frequencies at baseline (Figure 7B, left panels), the cross-correlograms acquire similar characteristics following the stimulation (Figure 7B, middle panels). This effect can be visualized by comparing the corresponding power spectrum before and after stimulation (Figure 7B, right panels). Thus, the shift in main oscillation frequency observed on single-cell level (Figure 6) is visible on IO-wide level, meaning that electrical stimulation can entrain the entire network to a single, prominent frequency. To visualize the temporal structure of IO-wide oscillations, we compared the STO phase relationships among the 4 clusters (schematically shown in Figures 2, 8A1) before and after electrical stimulation. Cross-power spectral density (CSD, Figure 8B) calculated from average cluster traces shows again an average frequency shift from 4 to 6 Hz after electrical stimulus. CSD value represents the similarity of frequency domains and phase shift of STO clusters. In the example shown, peak CSD increases 10 times after electrical stimulation while PSD only increased 2.8 times (Figure 7). This reflects the afore-mentioned frequency shift accompanied by an increased phase-locking of STOs between the different pixel clusters. Finally, point-by-point correlation matrix (Figure 8C) highlight the complexity of phase relationships between the 4 clusters. While the activity of some clusters is poorly correlated during baseline (for example C2-C3, Figure 8C1), they become strongly coupled after stimulus (as shown by correlation coefficients in Figure 8C2). The coupling can be either in-phase (C1-C2, C3-C4) or in anti-phase (C1-C3, C1-C4, C2-C3, C2-C4). Interestingly in this example, phase-coupled pairs of clusters reside in same subnuclear region of the IO (C1 and C2 are in IOPr while C3 and C4 in IOD).

## 4. Discussion

Taken together, the results presented here demonstrate that if introduced to brain tissue via stereotactic injection 7 days before *in vitro* experiments, A6+ can be used to monitor subthreshold membrane voltage events on single-cell and network level.

It should be noted that while a vast literature exists where important insights on network activity dynamics have been obtained with other voltage indicator dyes in brain structures other than the IO (see for example Zochowski et al., 2000; Bai et al., 2006; Chang et al., 2007; Momose-Sato et al., 2012; Jackson, 2013; Liang et al., 2015) but to our knowledge, no reports have been published of a method where subthreshold activity of ≤ 15 mV amplitude could be observed on single-cell and network level without trial averaging. We expect that using A6+ in combination with the *in vivo* pre-injection workflow will be also useful in investigation of network phenomena other than IO STOs.

Here, we will discuss the staining procedure as well as compare the results to those reported in earlier literature (Leznik et al., 2002; Leznik and Llinás, 2005).

### 4.1. ANNINE-6plus Labeling During the Post-injection Period

A6+ has been shown to be a pure electrochromic voltage-sensitive dye (Kuhn and Fromherz, 2003; Fromherz et al., 2008; Kuhn and Roome, 2019) with high sensitivity when excited at the red spectral edge of absorption (Kuhn et al., 2004; Kuhn and Roome, 2019). In earlier studies with ANNINE dyes, labeling was done by bolus loading (Kuhn et al., 2008) or electroporation of single Purkinje neurons (Roome and Kuhn, 2018). Here, we also use bolus loading to introduce the dye into the tissue. However, we choose to inject A6+ at a concentration above saturation so that small dye particles can be observed on D0 in the tissue (Figure 2E). To improve A6+ voltage imaging under this labeling conditions, it is advantageous to allow sufficient time for the dye to dissolve completely and to diffuse within the mesoscale structure. The excess dye from the particles and the long labeling time results in a larger volume of homogeneous labeling. Over time the dye bound to the extracellular leaflet of the plasma membrane will be washed out while internalized dye or dye that flipped from the outer membrane leaflet to the inner leaflet remain trapped. This is confirmed by the fluorescence intensity profiles obtained from fixed IO slices processed at D0, D1, and D7 post-injection. Also, the labeling becomes more homogeneous as dye particles dissolve and diffusion reduces the labeling gradients.

The dye trapped intracellularly labels all membrane surfaces. As voltage changes only occur over the plasma membrane, most of the trapped dye is expected to give a relatively constant level of background fluorescence with weak contribution to the membrane voltage signal. Indeed, the clearing of the extracellular dye and labeling of the intracellular leaflet of the plasma membrane is sufficient to allow voltage imaging with acceptable SNR. The notion that the signal is originating from the intracellular leaflet of the plasma membrane is supported by the observing the direction of the fluorescence change: a depolarization of an IO neuron, as confirmed by patch clamp experiments, results in a positive fluorescence change of A6+ (Figure 5A). If the dye would be located in the extracellular leaflet, the fluorescence change would be negative (Kuhn and Roome, 2019).

In summary, injecting A6+ in IO in living animal a week before acute slice experiments provides several advantages compared to bath application of A6+ in acute slices: (1) decrease in cell death in response DMSO application (see Supplementary Figure 2A); (2) recovery of cytoplasmic membrane quality allows improved success in *in vitro* experiments (see Figure 5) while the process of bath loading damages cell membranes (see Supplementary Figure 2B); (3) more homogenous labeling within the slice, as bath-applied A6+ penetrates slice tissue only up to 20 μm from surface (see Supplementary Figure 2D).

### 4.2. Spatial Resolution of Voltage Imaging During the Post-injection Period

To validate the utility of an extended labeling period, we examined whether these progressive changes in the anatomical distribution of A6+ are linked with improved signal quality using acute *in vitro* slice preparation. To this end, we measured the spatial distribution of the power spectral density across the known IO STO oscillation frequency band (3–12 Hz). Not surprisingly, we found that the broadening of uniformly-stained region (Figure 1) is accompanied by similar broadening of relatively uniform distribution of the oscillatory power (Figure 2C). This indicates that for high SNR voltage imaging, it is beneficial to allow the labeling to recede in strength while spreading into large volume.

On a cellular level, the internalization of A6+ and the clearing of extracellular A6+ during the 7 post-injection days (Figure 2) is accompanied by improved visibility of neuronal structures and enhancement of the resolution of STOs despite some dye remaining bound to intracellular structures (Figure 6). Notably, it is possible to find regions in slices prepared immediately after dye injection that contain neurons well-enough labeled so that STOs can be discerned (example shown in Figures 5B1,C1,E1). However, as we find no evidence for decrease in neuronal health nor in the network activity features during prolonged incubation period (evidenced by similar coherent oscillating cluster density identified in slices prepared immediately after dye injection as well as a week later), it is clearly recommended to allow longer labeling period whenever otherwise feasible. This is in line with previous findings that intracellular A6+ remains trapped in Purkinje neurons *in vivo* for weeks without affecting their health (Roome and Kuhn, 2018, 2019).

### 4.3. Subthreshold Oscillations in the Inferior Olive Can Be Visualized With ANNINE-6plus

As a proof-of-principle, we use the approach to replicate and extend classical observations from nearly two decades ago (Llinás and Yarom, 1986; Manor et al., 2000; Llinás et al., 2002) showing clustered subthreshold activity patterns in the inferior olive as well as the manner that electrical stimulation modifies the spatial organization of coherently oscillating clusters. The subthreshold oscillations of the inferior olive have been extensively studied using intracellular recordings from individual cells (Llinás and Yarom, 1986; Devor and Yarom, 2002a; Llinás et al., 2002). Reports from paired recordings in nearby neurons (Manor et al., 2000; Devor and Yarom, 2002a,b; Long et al., 2002) have indicated that the oscillations in most cases will be found to be phase-locked among neighboring neurons, but the spatial extent of phase coherence across the entire IO network has been unclear. As the size of neuronal groups oscillating synchronously are likely to be a relevant determinant of the emergence of synchronous activity in the cerebellar cortex, various anatomical and electrophysiological methods (Hoge et al., 2011; Lefler et al., 2013; Kølvraa et al., 2014; Vrieler et al., 2019) have been employed in attempts to delineate cluster characteristics. In addition to these indirect approaches, a handful of works (Devor and Yarom, 2002a,b; Leznik et al., 2002; Leznik and Llinás, 2005) have employed early voltage-sensor technology to directly visualize how the IO network is organized into coherently-oscillating clusters. While highly influential works, the low sensitivity of the recording technique necessitated stimulus-triggered averaging of trial data and could not reliably be used to investigate subthreshold activity on single-cell level.

Here, using more modern voltage-sensitive dye, optical equipment and analytic approaches, we broadly speaking replicate the main experiments presented in (Leznik et al., 2002), without the need to use trial averaging to resolve spatial and temporal features of the STO.

Based on the spatial and temporal features of the voltage oscillations recorded optically we conclude that they represent the IO STO activity. Considering that cell density in IO is about of 3 ± 2 cells in a 50.50.25 μm volume (Devor and Yarom, 2002a; Vrieler et al., 2019), A6+ injections within IO give us the ability to perform voltage imaging from an area covering a population of 1,209 ± 806 neuronal units and sort spontaneously active neurons in clusters of 8 ± 5 neurons.

Importantly, the staining method described here leads to a high-enough SNR to allow clear resolution of STOs not only as averages from the coherent clusters, but also on much finer spatial scale. As shown in Figure 6, voltage oscillations with the characteristics of STOs can be discerned from small subcellular regions. While the resolving power of the optical system in our use is limited, the results demonstrate the conceptual possibility of investigating IO STOs without investing in high-end laser-scanning imaging systems or establishing genetically-encoded voltage imaging methodology, often prohibitively complicated and time-consuming. Furthermore, while a single-injection staining might result in a prohibitively small labeled volume, this can be improved by injecting at several locations in the same structure.

Notably, while we were not specifically examining subthreshold events other than the rather slow STOs, in some trials with high frame rate (120 Hz) we also noted that the electrical stimulation of glutamatergic afferents reliably evoked faster A6+ fluorescence signal transients localized on small structures (around 1–2 μm size; see Figure 6E2). The waveform of these events in response to trains of stimulations at up to 100 Hz suggested they represent synaptic depolarization events. Thus, with some improvements in the spatiotemporal resolution in optical system, A6+ has the potential to be used as a tool in investigating synaptic interactions and plasticity within a network.

### 4.4. Modulation of IO STOs by Electrical Stimulation

Having established the staining procedure leading to optimal voltage imaging conditions, we briefly examined how electrical stimulation of afferent, excitatory axons modifies the IO STOs on network and single-cell level. We found that in line with previous reports (Leznik et al., 2002; Lefler et al., 2013), stimulation can enhance oscillations in IO slice on both network (Figure 5E) and single-cell (Figure 6E) level. In cases where STO activity was present on network or single-cell level prior to stimulation, we observed a clear increase in oscillation coherence among clusters (Figure 5E2), even across subnuclear boundaries (note the distribution of clusters in various parts of the IO in Figures 3, 4). Furthermore, in all cases, this was driven by emergence of an oscillation with an identical frequency across the clusters. Importantly, the emerging frequency varied between slices (3–7 Hz; data not shown) but it did not match with stimulation frequency. This suggests that the resulting frequency depends on the specific physiological features and connection strengths of the oscillating units, in line with previous theoretical and experimental suggestions (Torben-Nielsen et al., 2012; Lefler et al., 2013).

Strikingly, the cross-covariance of the network activity showed that even though the stimulation-evoked oscillation frequency was identical across the entire IO, it was not phase-aligned. This suggests that the long-hypothesized, IO-wide stable phase lags between oscillating groups of neurons (Jacobson et al., 2009; Torben-Nielsen et al., 2012; Lefler et al., 2013) may be discernible with the A6+ voltage imaging methodology.

## 5. Summary

The results presented here demonstrate the utility of the voltage-sensitive dye ANNINE-6plus in monitoring subthreshold activity on network as well as cellular level. We expect that the method developed here can be applied with minor modifications to many other brain regions or nuclei. The long-lasting labeling allows also chronic preparations with fibers or GRIN lenses.

Importantly, even though the IO STOs can be larger in amplitude and duration than other commonly-studied subthreshold events (such as post-synaptic potentials), A6+ imaging could potentially be applicable to studying synaptic interactions on network level.

While we were not aiming here to deeply investigate the network-level oscillatory behavior in IO, the data obtained with A6+ imaging suggests several insights in this topic. First, the emerging clusters of coherently oscillating cells are similar in size as have been proposed previously. This strengthens the notion that the connectivity within the IO is non-homogeneous and rather organized in a mesh of tightly-coupled clusters of synchronously-active neurons.

Second, the electrical stimulation entrains wide regions of the IO into an oscillation with identical peak frequency, without strict phase alignment. Interestingly, the resulting frequency does not reflect stimulation frequency, and it varies between slices. This indicates that emerging oscillation is not a simple outcome of entrainment of the network with stimulation; rather, the resulting oscillation frequency is subtly dependent on the intrinsic properties of individual neurons involved in the oscillation.

## Data Availability Statement

The datasets presented in this study can be found in online repositories. The names of the repository/repositories and accession number(s) can be found below: https://github.com/oist/Frontiers_ANNINE-6plus_IO_STO/tree/master/data.

## Ethics Statement

The animal study was reviewed and approved by Okinawa Institute of Science and Technology (OIST) Graduate University Institutional Animal Care and Use Committee (IACUC). OIST is an Association for Assessment and Accreditation of Laboratory Animal Care (AAALAC International) accredited facility.

## Author Contributions

KD, BK, and MU designed the research and wrote the paper. KD and MU performed the research and analyzed the data. All authors contributed to the article and approved the submitted version.

## Conflict of Interest

The authors declare that the research was conducted in the absence of any commercial or financial relationships that could be construed as a potential conflict of interest.

## Supplementary Material

Supplementary Figure 1 Sparse eGFP labeling and A6+ staining of IO neurons. **(A1,A2)** Two examples of sparse eGFP labeling of IO neurons taken with 5x objective. IO subnuclei boundaries are indicated with dashed lines. **(B)** 40x confocal image of two IO neurons labeled with eGFP. **(C)** Two confocal images of A6+ staining of IO at D0 (left) and D7 (right). White arrows denote A6+ inclusions inside the cells while black asterisk within shaded regions delineate myelinated fiber tracts.

Supplementary Figure 2 Bath application of A6+, DMSO3% on brainstem slices. **(A)**. The quality of experiments where IO neurons (IOn) are current-clamped on brainstem slices in relation to DMSO-containing A6+ staining solution incubation time. Orange curve is a second-order polynomial regression for the 10 example slices. **(B)** A6+ staining quality (ratio between A6+ staining and DAPI staining) in relation to patch-score for the same 10 slices. **(C)** A6+ staining quality is represented in function of time spent in DMSO 3%, A6+ solution. Note that obtaining adequate staining (with staining quality larger than 1) is prohibitively damaging IO neurons. **(D)** Normalized A6+ fluorescence in relation to tissue depth for slices where A6+ staining quality is more than 1. Inset shows an orthogonal view of a IO slice incubated in A6+ solution (black arrow denotes tissue surface).
