# Supplementary figures and images for: Imaging Subthreshold Voltage Oscillation With Cellular Resolution in the Inferior Olive in vitro
Source: Front Cell Neurosci. 2020 Dec 14;14:607843. doi: 10.3389/fncel.2020.607843 (PMC7767970; doi:10.3389/fncel.2020.607843)

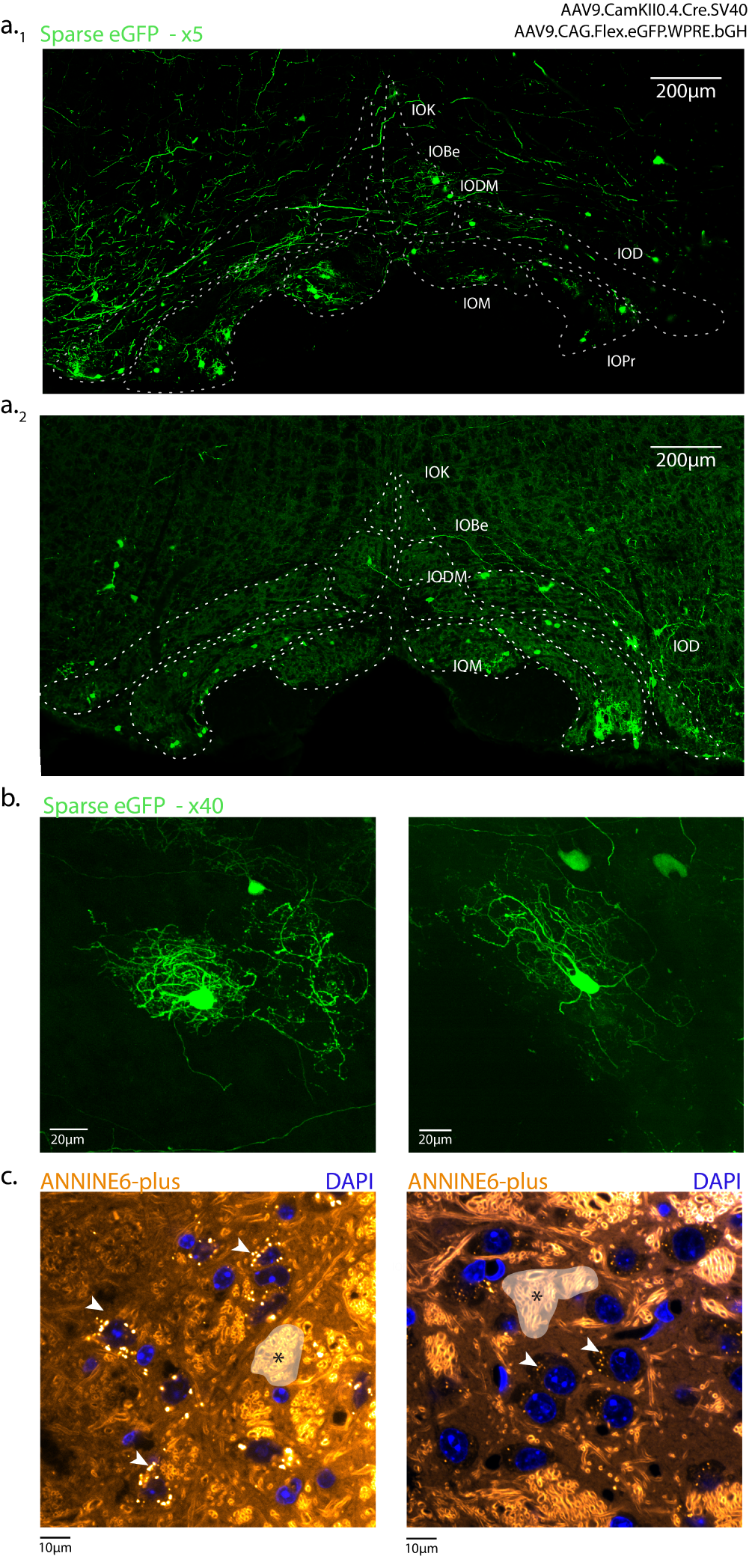

Supplement: Supplementary Figure 1 — Sparse eGFP labeling and A6+ staining of IO neurons. (A1,A2) Two examples of sparse eGFP labeling of IO neurons taken with 5x objective. IO subnuclei boundaries are indicated with dashed lines. (B) 40x confocal image of two IO neurons labeled with eGFP. (C) Two confocal images of A6+ staining of IO at D0 (left) and D7 (right). White arrows denote A6+ inclusions inside the cells while black asterisk within shaded regions delineate myelinated fiber tracts. [file Image_1.TIF]

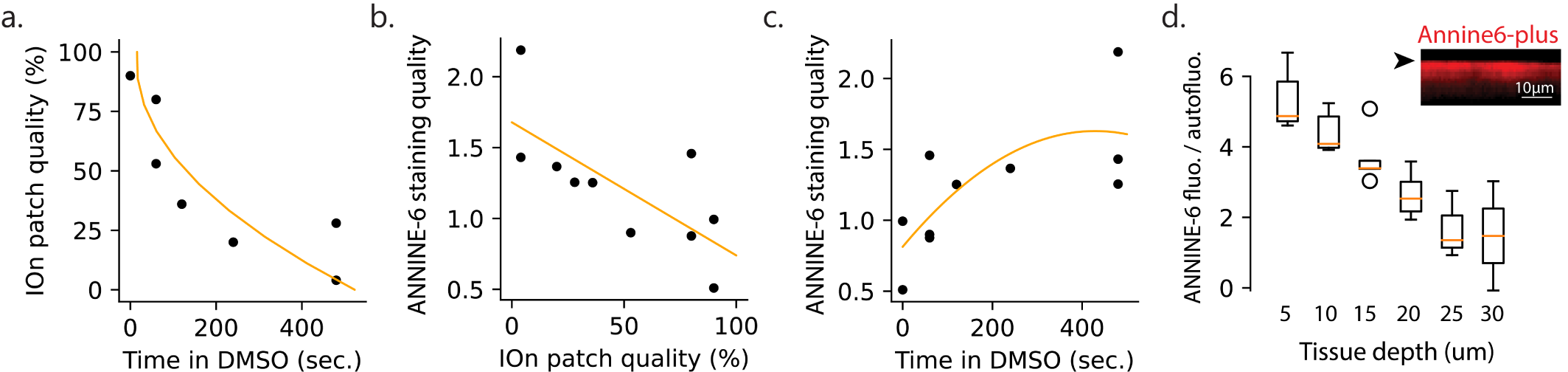

Supplement: Supplementary Figure 2 — Bath application of A6+, DMSO3% on brainstem slices. (A). The quality of experiments where IO neurons (IOn) are current-clamped on brainstem slices in relation to DMSO-containing A6+ staining solution incubation time. Orange curve is a second-order polynomial regression for the 10 example slices. (B) A6+ staining quality (ratio between A6+ staining and DAPI staining) in relation to patch-score for the same 10 slices. (C) A6+ staining quality is represented in function of time spent in DMSO 3%, A6+ solution. Note that obtaining adequate staining (with staining quality larger than 1) is prohibitively damaging IO neurons. (D) Normalized A6+ fluorescence in relation to tissue depth for slices where A6+ staining quality is more than 1. Inset shows an orthogonal view of a IO slice incubated in A6+ solution (black arrow denotes tissue surface). [file Image_2.TIF]
